# Shared decision-making in medication management: development of a training intervention

**DOI:** 10.1192/pb.bp.116.053819

**Published:** 2017-08

**Authors:** Ute Stead, Nicola Morant, Shulamit Ramon

**Affiliations:** 1 Cambridgeshire and Peterborough NHS Foundation Trust, Cambridge; 2 University College London, London; 3 University of Hertfordshire, Hatfield

## Abstract

Shared decision-making is a collaborative process in which clinicians and patients make treatment decisions together. Although it is considered essential to patient-centred care, the adoption of shared decision-making into routine clinical practice has been slow, and there is a need to increase implementation. This paper describes the development and delivery of a training intervention to promote shared decision-making in medication management in mental health as part of the Shared Involvement in Medication Management Education (ShIMME) project. Three stakeholder groups (service users, care coordinators and psychiatrists) received training in shared decision-making, and their feedback was evaluated. The programme was mostly well received, with all groups rating interaction with peers as the best aspect of the training. This small-scale pilot shows that it is feasible to deliver training in shared decision-making to several key stakeholders. Larger studies will be required to assess the effectiveness of such training.

Shared decision-making is a collaborative process in which clinicians and patients make treatment decisions in partnership. Both partners bring valuable contributions to this process: patients share their experiences, values and preferences, and clinicians support patients in clarifying these, as well as providing clinical expertise and evidence-based information about treatment options. Jointly, they aim to reach an agreement on the best way to proceed.^1–3^

Shared decision-making is considered particularly relevant for preference-sensitive decisions, where there are several reasonable treatment options and evidence does not support a clear best choice. Different options require the balancing of possible benefits against potentially significant adverse or as yet unknown effects.^3–5^ Most medication decisions in mental health fall into this category, which makes psychiatric medication management an important area for shared decision-making.^6^

There are good reasons for encouraging adoption of shared decision-making in mental health. Many patients wish for greater participation in treatment decisions.^7^ In chronic conditions, where long-term healthcare decisions are required, studies have shown that shared decision-making improves satisfaction, adherence and well-being.^8^ Shared decision-making is at the core of recovery principles which promote autonomy and self-management skills, as well as being considered essential for delivering patient-centred care.^9^ National and international government initiatives,^1,10–13^ such as the UK Health and Social Care Act 2012 and the US Patient Protection and Affordable Care Act 2010, endorse shared decision-making, as do professional bodies^14–16^ and practice guidelines.^17–21^ Shared decision-making is an expected element of all NHS care (Health and Social Care Act 2012, s. 23, 26). Although there is evidence of its benefits, and many patients want greater involvement,^7^ the adoption of shared decision-making into routine clinical practice continues to be slow,^22,23^ especially in mental health ^24^

A range of interventions has been developed to promote implementation of shared decision-making, predominantly in physical health.^25–28^ Although this is an evolving area, relatively few interventions focus specifically on treatment decisions in mental health.^6,29–33^ Evidence is sparse regarding the effectiveness of such interventions in general,^23,25^ and in mental health settings in particular,^33^ although some promising results have been reported. These include a study of in-patients with schizophrenia or schizoaffective disorder who received five sessions of shared decision-making training;^29^ a multifaceted programme based on shared decision-making concepts for primary care physicians treating patients with depression;^30^ a peer-run Decision Support Centre in the waiting area of a psychiatric medication clinic;^31^ and online resources supporting shared decision-making.^32^ The limited evidence available points towards interventions being more effective when involving healthcare professionals and patients together, rather than only targeting one group.^23^

The Shared Involvement in Medication Management Education (ShIMME) project was set up to promote shared decision-making of service users (the term ‘service users’ was used in the ShIMME project and has been retained here) in medication decisions by delivering a specially developed training programme to three key stakeholder groups: service users, care coordinators and psychiatrists. (In the context of the ShIMME project ‘care coordinator’ refers to psychiatric nurses, social workers, occupational therapists, psychologists, support workers, peer workers and students training in these disciplines.) To our knowledge this is the first UK-based project to deliver and evaluate such an intervention that targets multiple stakeholders simultaneously.

The project was a partnership between Cambridgeshire and Peterborough NHS Foundation Trust (CPFT) and Anglia Ruskin University. Reflecting the strong collaborative ethos, service users were active team members jointly with academic researchers, mental health practitioners and other professionals working within CPFT.

This paper describes the stages of the ShIMME project: consultations about shared decision-making, development and implementation of a pilot intervention, and evaluation of feedback. It is one of several articles relating to the project.^34–36^

## Method

### Consultation phase

The initial phase of the project involved a literature review and consultation with local stakeholders about the process of shared decision-making. Data were collected via focus groups with practitioners and users of adult mental health services in CPFT. Four focus groups were conducted with service users (*n* = 27), two with psychiatrists (*n* = 4), one with community psychiatric nurses (CPNs, *n* = 10), and one with care coordinators other than CPNs (*n* = 8). Four individual telephone interviews were also conducted with psychiatrists. Discussion was generated in response to open questions about current practice in medication management, how decisions should ideally be made, perceived barriers to and facilitators of shared decision-making, and how shared decision-making training should be conducted. Consultation groups lasted around 90 min and were audio recorded. The anonymised transcripts were analysed using thematic analysis,^37^ conducted with NVivo software (www.qsrinternational.com). This involved a detailed exploration of transcript texts by two team members who worked in collaboration to iteratively develop themes.

The following themes about shared decision-making in medication management, which are presented in more detail elsewhere,^34^ emerged from this analysis and fed into the development of the training programmes.

1. Ongoing respectful, trusting, open and honest relationships are paramount – service users' concerns and experiences need to be heard and taken seriously.
2. Differences of power in the consultation can be complex and a barrier to shared decision-making – clinicians can underestimate the effect this has.
3. Access to reliable, user-friendly information is essential, including information about reducing or coming off medication and adverse effects of medication.
4. All available treatment options should be considered, including non-pharmacological treatments.
5. The process of shared decision-making needs to be flexible, taking into account preferences and situations which may change over time. Acute stages of illness or crisis situations were identified as times when shared decision-making would be likely to be more problematic.
6. Broader stakeholders (beyond service user and prescribe!^1^) have important roles in the shared decision-making process (e.g. other professionals, carers).
7. There is currently significant variation in medication management and the extent to which this involves shared decision-making.

### Training intervention: design

A multidisciplinary working group including service users, academic researchers, psychiatrists, a mental health nurse and a pharmacist met regularly to develop the training intervention. The results of the consultations, literature review and examples of existing practice fed into the development.

Training was designed to be delivered to service users, care coordinators and psychiatrists in parallel but separate groups. The aim was to optimise the impact of the intervention by delivering it simultaneously to key stakeholders who are actively involved in medication management, while addressing the specific training needs and concerns of each group. Each group was facilitated by a service user trainer, and either a psychiatrist (for service user and psychiatrist groups) or a mental health nurse (for care coordinator groups), allowing participants to learn from two relevant perspectives.

The programme employed a range of interactive learning methods. These included specially commissioned video material showing different clinical scenarios, small group exercises, general group discussions, use of testimonials and role plays. The resource materials and hand-outs covered a diverse range of views and approaches, to raise awareness and stimulate discussion. All participants had access to the public section of the project website (www.shimme.arcusglobal.com) as well as a secure discussion forum for their group.

The training programmes for the three stakeholder groups covered the same core content:

- background to the project
- key components of shared decision-making in the clinical encounter and rationale for promoting shared decision-making
- barriers to and facilitators of shared decision-making
- awareness of the effects of power imbalances in psychiatric consultations
- developing collaborative relationships
- importance of clarifying personal preferences, values and experiences in shared decision-making
- the concept of a ‘meeting of two experts’ in the clinical encounter, with personal experience and clinical expertise complementing each other^38^
- accessing and appraising information about medication, including examples of decision aids
- raising awareness of adjuncts or alternatives to medication
- addressing issues around coming off or reducing medication
- trialling of versions of three paper-based tools developed for supporting and recording the shared decision-making process
- information about useful websites.

In addition to the core content, the service user groups focused on:

- practising setting personal goals and identifying preferences
- making use of a personal well-being plan and self-help resources
- looking beyond medication to enhance well-being, drawing on Deegan's work on ‘personal medicine’^39^
- introduction to assertiveness
- how to access information about medication, including a talk by a National Health Service (NHS) trust mental health pharmacist, who was available for further discussion afterwards
- supported ‘hands-on’ experience exploring relevant websites.

Besides the core content, the care coordinator training focused on adopting the role of a ‘shared decision-making coach’, supporting service users to play a more active part in the shared decision-making process ^35^ The programme for psychiatrists focused on competencies and resources that support embedding shared decision-making into routine clinical practice while acknowledging real-life challenges.

### Training intervention: delivery

All training group participants were recruited from CPFT community mental health services: service users from the rehabilitation and recovery pathways; and professionals from these services and from assertive outreach teams. Service users were invited to participate by their care coordinators and psychiatrists and care coordinators were approached by team managers. In total, 47 service users, 12 psychiatrists and 35 care coordinators took part in the training.

Training was held in three different locations to reduce travelling for participants. Service users were reimbursed for travelling costs and received a fee (£40) for completing an evaluation before and after the programme.

The training was delivered in small group settings (2–12 participants), with each cohort completing their course of training together. An atmosphere of trust, acceptance and respect was encouraged. Participants had the opportunity for informal interaction before and after sessions as well as during breaks. Facilitators and project team members could be contacted between sessions for additional support.

Service user training groups were structured into four 2h sessions, meeting fortnightly After the training, two follow-up sessions were offered for ongoing support. Care coordinators met three times, at monthly intervals, for 1.5 h. Psychiatrists had two 2 h sessions, one month apart, with an online self-study component. For organisational reasons one multidisciplinary team of clinicians received their training together in a single day.

### Evaluation

The experience and impact of the training intervention was evaluated by collecting quantitative and qualitative data anonymously from participants at different stages of the project.

After providing baseline data, participants and facilitators completed a short questionnaire after each session, and participants completed a longer one immediately after the final training session. The questionnaires explored what the participants had hoped to learn from the programme, their views on its content and impact, and feedback on particular sessions, practical aspects, teaching methods and support materials.

Analysis of quantitative and qualitative data examining the impact of the intervention at a 12-month follow-up, as well as an economic analysis, will be reported on separately.

## Results

Although care coordinators and psychiatrists were mostly trained in separate groups, their demographic and feedback data are reported as one group of clinicians. Demographic and attendance data for service users and clinicians are shown in Table 1 and Table 2.

**Table 1:** Demographic characteristics of participants

	Service users (*n* = 47) *n* (%)	Clinicians (*n* = 47) *n* (%)
Female	22 (47)	33 (70)

Male	25 (53)	14 (30)

Mean age, years	48	45

Ethnicity		
White	42 (89)	37 (79)
Black	1 (2)	1 (2)
Asian	0	4(9)
Other	3 (6)	2 (4)
No data	1 (2)	3 (6)

Education		
Tertiary/further	30 (64)	
Secondary	14 (30)	
Primary or less	1 (2)	
No data	2 (4)	

Employment^a^		
Paid/self-employed	3 (6)	
Voluntary employment	7 (14)	
Unemployed	25 (50)	
Student (including part-time)	4 (8)	
Age-related retirement	4 (8)	
Other	7 (14)	

Professional background of clinicians		
CPN/nurse		11 (23)
Occupational therapist		9 (19)
Clinical psychologist		2 (4)
Social worker		2 (4)
Support time and recovery worker		6 (13)
Peer support worker		2 (4)
Team leader/deputy manager		3 (6)
Psychiatrist		12 (26)

CPN, community psychiatric nurse.

a. More than one answer possible.

**Table 2:** Session attendance

	Patients	Care coordinators	Psychiatrists
Sessions offered	4 × 2 h	3 × 1.5 h	2 × 2 h

Cohorts training delivered to	6	2 + 1 (team training day)	2 + 1 (team training day)

Attendance	37 (79%) attended at least 3 sessions of 4	20 of 21 (95%) attended at least 2 sessions of 3 14 attended team training day	6 of 10 (60%) attended both training sessions 2 attended team training day

The mean length of contact with mental health services for service users was 17 years. The most common reported diagnoses were schizophrenia, schizoaffective disorder or psychosis (*n* = 28, 60%), followed by depression (*n* = 12, 26%), bipolar affective disorder (*n* = 9, 19%), personality disorder (*n* = 5,11%), anxiety (*n* = 4, 9%) and post-traumatic stress disorder (*n* = 4, 9%). Some participants reported multiple diagnoses. The majority of service users received state benefits (*n* = 43, 92%), with *n* = 39 (83%) on a disability living allowance.

Immediate post-programme feedback was given by 61 (65%) participants: 33 (70%) service users and 28 (60%) clinicians, including 22 (63%) care coordinators and 6 (50%) psychiatrists. Before starting the programme, service users mostly hoped to learn about ways to cope with their symptoms not solely focused on medication, to understand their medication better and to negotiate decisions. Clinicians were particularly interested in improving their practice, learning about the model and process of shared decision-making, availability of support materials, and sharing experiences with colleagues.

Expectations of the programme were largely met in both groups, with the majority of participants expressing a positive view about its content. In all groups, the opportunity for discussion and exchange of views with peers was highlighted as the best aspect of the programme. In addition, service users valued the clarity of the information conveyed, access to resources and the prospect of greater collaboration in consultations. Clinicians also appreciated access to resources and the information given, as well as the opportunity to reflect on their own practice, particularly in the case of psychiatrists.

There was little negative feedback. Just over half of service users (*n* = 17, 52%) and the majority of clinicians (*n* = 20, 71%) did not identify any aspects of the programme as being ‘least satisfactory’. Some service users mentioned dissatisfaction with practical aspects or teaching methods, and a few referred to difficulties reading all the paperwork/understanding everything. A small number of psychiatrists expressed concerns about a perceived bias against their profession. Most participants felt the training was pitched at the right level. Use of the project website was variable, with about half of service users visiting it outside sessions. Most psychiatrists visited the website, but only a few care coordinators did. The online forum was not used by any of the groups.

Most clinicians rated the training programme as relevant to their clinical practice, but fewer expected that what they had learned would shape their future practice. Over half of service users expected or were at least hopeful that the programme would affect future practice.

A summary of the post-programme feedback is given in Table 3.

**Table 3:** Summary of feedback

	Service users (*n* = 33)	Clinicians (*n* = 28)
Most important things participants hoped to learn^a^	Lifestyle changes/coping with symptoms/alternatives to medication Understanding medication Medication management/SDM/negotiating decisions Sharing experiences Understanding side-effects of medication Assertiveness/confidence with professionals Info about project/research Reducing/coming off medication	Improving practice Learning about SDM model and process Support materials/tools for SDM Sharing ideas and practice Information about medication, including side-effects and coming off Learning about the project Gaining confidence in discussions with service users Understanding service user perspective

Views on content of the programme	Positive views 28 (85%): interesting, helpful, informative, empowering, encouraging, learned a lot Other comments 4 (12%): SDM needs to be implemented from consultant psychiatrist downwards/did not learn that much concrete	Positive views 21 (75%) 18 (82%) care coordinators, 3 (50%) psychiatrists: very good, good, interesting, informative, well-balanced Other comments 4 (14%): repetitive, some prejudice against psychiatrists

Best aspects of programme^a^	Meeting others, exchanging views and experiences, supportive environment Information conveyed, new ideas and access to resources Learning to be involved in my medication management, feeling confident my views will be listened to	Interaction with others, chance to discuss implementation of SDM Direction regarding resources/tools to support SDM, information Concept of SDM Gaining confidence in promoting SDM/putting SDM into practice Reflecting on own practice Getting service user perspective

Least satisfactory aspects^a^	Practical aspects, teaching methods Not understanding everything, not able to read all paperwork Parts boring, same	Practical aspects, teaching methods Perceived bias against psychiatrists Did not improve personal knowledge of medication Content

Training pitch at right level	32 (97%)	20 (71%): 17 (77%) care coordinators, 3 (50%) psychiatrists

Use of project website	17 (52%)	11 (39%): 6 (27%) care coordinators, 5 (83%) psychiatrists

Relevance of training programme and impact on future practice	Expecting impact: 12 (36%) Hopeful of impact: 7 (21%) Doubtful/unsure: 5 (15%) Relevant for others: 2 (6%)	Relevant: 23 (82%) Impact on own practice in future: yes 16 (57%), no 1 (4%), hopeful/probably 2 (7%)

SDM, shared decision-making.

a. Listed in order of frequency.

## Discussion

The ShIMME project was a small-scale exploratory project with an emphasis on service users co-leading in all aspects, while aiming to ensure the views of all key stakeholders were integrated into the development and delivery of the training intervention.

The training programme was well received overall, demonstrating the feasibility of providing group-based training in shared decision-making to service users and practitioners in NHS community settings. In this case, service user participants were drawn from the rehabilitation and recovery pathways which serve people with chronic and often severe mental health problems. Demographic data from participants indicated high levels of chronicity and disability. The positive feedback, good attendance and engagement from this group suggest that taking part in shared decision-making training is possible and worthwhile for people experiencing a range of mental health challenges.

Feedback indicated that service user participants were interested in being actively involved in managing their mental health, including gaining a better understanding of medication and exploring a range of other strategies to foster well-being. Clinicians showed an interest in improving their practice by learning about shared decision-making.

Members of all the stakeholder groups gave positive feedback about the group-based training, allowing for the exchange of ideas and experiences with peers. This was also reflected in facilitator comments about the supportive atmosphere and participants' enjoyment of meeting with each other in the service user groups. Interaction with peers seemed to be an important aspect of the whole programme.

There may also be advantages in service users and clinicians attending joint training groups, allowing participants from different backgrounds to work together on an equal basis and to gain a better understanding of others' perspectives without the pressures and structures of the clinical encounter. The involvement of carers and important others might bring further benefits.

The feedback about the content, approach and pitch of the teaching within the group of psychiatrists was not quite as positive as in the other groups. The reasons for this are likely to be multifaceted and would warrant further exploration, with possible adjustments of the programme. Away to enhance acceptability and engagement would be to encourage more psychiatrists to become involved in shared decision-making training and development of tools.^28^ Use of the project website was limited, in particular by care coordinators and service users. Technical difficulties with the website might have contributed to this, but comments during sessions indicated that some participants, particularly service users, had low IT confidence and limited internet access outside the training sessions. Future training programmes will need to provide non-digital resources, as well as supporting access and use of IT resources.

The project team developed three paper-based tools to support the process of shared decision-making, which were trialled in training groups and repeatedly revised. Although useful, these would need to be integrated into the existing electronic records system to be truly effective in promoting, supporting and documenting the process of shared decision-making without significantly affecting consultation time. At present this remains a challenge, but there have been some promising recent developments.^40^

Both groups of clinicians considered the training relevant to their clinical practice, although they appeared uncertain whether the programme would influence their future practice. This might be due to concerns about additional barriers to implementation or aspects of the training itself. Despite their positive feedback about the programme, service users were also cautious about its impact. This might reflect the perception that they have little influence in making significant changes to their healthcare delivery or doubts about positive initiatives being translated into clinical practice.

While this pilot programme had the limitations of a modest number of participants, not all of whom provided feedback, the consultation data from local stakeholders, the development of the training programme and the feedback from participants were all encouraging. Drawing on experiences from this project, CPFT has been working towards implementing shared decision-making across the trust by embedding shared decision-making into its procedures, raising awareness and offering training to practitioners across the trust (www.promise.global/sdm.html). The associated Recovery College (www.cpft.nhs.uk/about-us/recovery-college-east.htm), which is open to service users, family, friends and staff, also included training in shared decision-making. The pilot project benefitted from a supportive environment within the trust, and the success of implementation in other organisations would depend on their own local conditions.

The complexity and difficulty of implementing shared decision-making in a mental health setting should not be underestimated.^36,41^ For it to truly become a routine part of clinical practice, changes in attitudes and behaviours are necessary among all parties involved, as well as the wider society.^36,42^ Psychiatrists are well placed to take on a leadership role in promoting shared decision-making within health services and should also be pivotal in explaining the benefits of increased patient autonomy and responsibility to the general community.
